# Solubilizer-Assisted Formulation of Porphyrin-Containing Lipid Nanoparticles for Photodynamic Applications

**DOI:** 10.3390/nano16201329

**Published:** 2026-10-20

**Authors:** Yumi Kimura, Rina Naganawa, Yuta Takano, Masatoshi Maeki, Manabu Tokeshi, Yusuke Sato, Yuma Yamada

**Affiliations:** 1 Laboratory for Molecular Design of Pharmaceutics, Faculty of Pharmaceutical Sciences, Hokkaido University, Kita 12 Nishi 6, Kita-ku, Sapporo 060-0812, Japan; kimura.yumi.f9@elms.hokudai.ac.jp (Y.K.); rinanaga1004@gmail.com (R.N.); y_sato@pharm.hokudai.ac.jp (Y.S.); 2 Research Institute for Electronic Science, Hokkaido University, Kita 20 Nishi 10, Kita-ku, Sapporo 001-0020, Japan; tak@es.hokudai.ac.jp; 3 Division of Applied Chemistry, Faculty of Engineering, Hokkaido University, Kita 13 Nishi 8, Kita-ku, Sapporo 060-8628, Japan; m.maeki@eng.hokudai.ac.jp (M.M.); tokeshi@eng.hokudai.ac.jp (M.T.)

**Keywords:** porphyrin, aggregation, photodynamic therapy, photosensitizer, mitochondria, MITO-Porter, microfluidics, nanoparticle

## Abstract

Hydrophobic porphyrin photosensitizers can lose photodynamic activity when densely loaded into nanoparticles because intermolecular aggregation promotes excited-state quenching. We investigated whether CAOM, a cinnamic-acid-derived oily solubilizer, could regulate the intraparticle organization of the π-extended porphyrin photosensitizer rTPA in MITO-Porter nanoparticles. Low-loading MITO-Porter (Low-MP), high-loading MITO-Porter (High-MP), and CAOM-containing High-MP (High-MP+CAOM) were prepared using the iLiNP microfluidic device. At an equivalent rTPA concentration, High-MP generated substantially less singlet oxygen than Low-MP, whereas High-MP+CAOM showed singlet oxygen generation comparable to Low-MP. High-MP+CAOM was internalized by HeLa cells and was observed in proximity to mitochondria. At comparable total lipid concentrations calculated from formulation inputs, High-MP+CAOM produced the greatest light-dependent reduction in cell viability, while dark toxicity remained limited under the tested conditions. Fluorescence spectroscopy was consistent with attenuated aggregation-caused quenching (ACQ), while cryo-transmission electron microscopy showed a more homogeneous intraparticle morphology after CAOM incorporation. These findings suggest that CAOM incorporation may support the photodynamic performance of porphyrin-containing lipid formulations under the tested conditions.

## 1. Introduction

Photodynamic therapy (PDT) induces local cytotoxicity through the light-triggered generation of reactive oxygen species (ROS), including singlet oxygen, by a photosensitizer [1,2,3]. Because photochemical reactions are confined to the irradiated region, PDT can provide spatially controlled treatment [4,5]. Mitochondria are attractive subcellular targets for PDT because mitochondrial oxidative damage can initiate apoptosis and may provide a mechanism that differs from that of anticancer agents primarily acting on nuclear DNA [6,7,8,9].

Previously, we developed the MITO-Porter, a liposome-based nanocarrier designed for mitochondrial delivery [10]. Building on this platform, we have explored the microfluidic formulation of photosensitizer-loaded MITO-Porters for mitochondria-targeted PDT.

The photosensitizer rTPA is a highly hydrophobic π-extended porphyrin derivative with near-infrared absorption properties [11]. Many porphyrin and phthalocyanine photosensitizers have planar aromatic structures and limited aqueous solubility, which can promote molecular aggregation and aggregation-caused quenching (ACQ) in aqueous media or within nanocarriers [12,13]. One strategy for addressing this limitation is the introduction of water-solubilizing substituents into the photosensitizer structure [14]. Laserphyrin, which is already in clinical use, is a representative example of this approach [15]. A complementary strategy is formulation-based solubilization, in which the photochemical properties of the photosensitizer and its delivery characteristics are optimized separately.

In previous studies, rTPA-loaded MITO-Porters showed light-dependent cytotoxicity in HeLa and SAS cells and antitumor activity in SAS xenograft models [11,16]. To increase the rTPA loading per unit lipid, we prepared a high-loading formulation (High-MP; drug-to-lipid ratio, 2.5 mol%), containing five times the nominal rTPA loading of the conventional Low-MP formulation (0.5 mol%). Unexpectedly, High-MP showed lower photodynamic activity than Low-MP. Transmission electron microscopy and small-angle X-ray scattering suggested that altered rTPA organization and aggregation within High-MP contributed to this loss of activity [17]. Thus, increasing photosensitizer loading without controlling its intraparticle molecular state may compromise the functional performance of the formulation.

**Figure 1:**
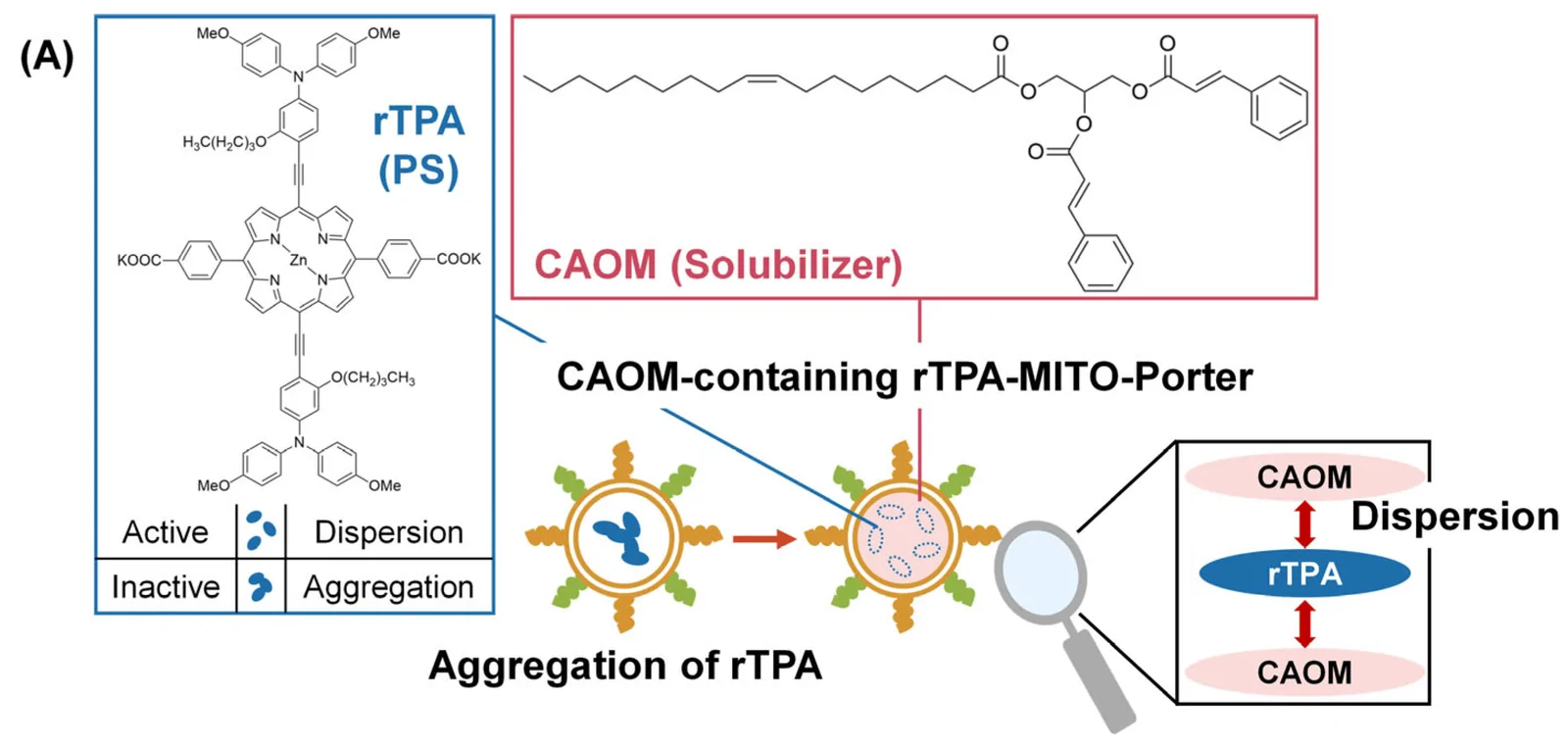

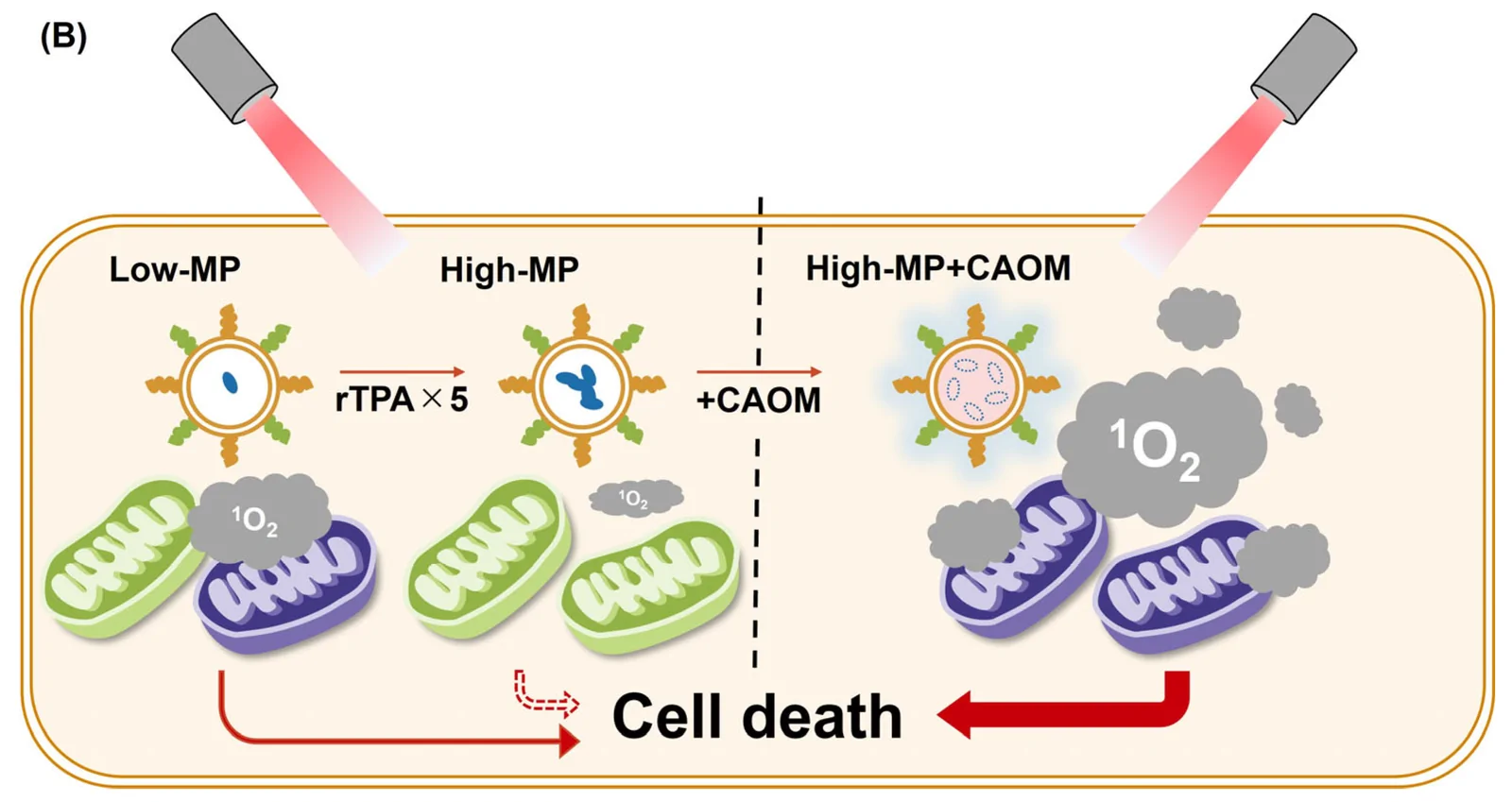
Schematic illustration of the study concept. (**A**) Proposed formulation strategy using CAOM to modulate the intraparticle organization of rTPA. (**B**) Conceptual comparison of Low-MP, High-MP, and High-MP+CAOM. The central dashed line distinguishes between the presence and absence of CAOM, while the arrow types and thicknesses schematically illustrate differences in their effects on cell death. This schematic is intended for conceptual illustration and does not represent quantitative differences. High rTPA loading without CAOM was associated with reduced photodynamic activity in our previous study, and CAOM incorporation was examined as a formulation strategy to attenuate this loss of activity. The molecular interactions and rTPA aggregation states depicted are hypothetical and have not been directly established in this study.

CAOM is an oily solubilizer derived from cinnamic acid that was developed to increase the apparent solubility of hydrophobic aromatic compounds, including curcumin and paclitaxel [18]. Aromatic interactions between CAOM and hydrophobic drug molecules have been proposed to contribute to its solubilizing effect. We therefore hypothesized that CAOM incorporation would modulate rTPA-rTPA interactions within High-MP and support high loading without the marked loss of photodynamic activity (Figure 1A). The conceptual differences between Low-MP, High-MP and High-MP+CAOM are illustrated in Figure 1B. In this study, we prepared CAOM-containing rTPA-loaded MITO-Porters using a microfluidic device and evaluated their physicochemical properties, singlet oxygen generation, cellular behavior, light-dependent cytotoxicity, fluorescence characteristics, and internal morphology.

## 2. Materials and Methods

### 2.1. Materials

1,2-dioleoyl-sn-glycero-3-phosphoethanolamine (DOPE) and N-(Methylpolyoxyethylene oxycarbonyl)-1,2-distearoyl-sn-glycero-3-phosphoethanolamine (DSPE-PEG2000) were obtained from NOF Corporation (Tokyo, Japan). Cholesterol and 1,2-dioleoyl-sn-glycero-3-phosphoethanolamine-N-(7-nitro-2-1,3-benzoxadiazol-4-yl) (ammonium salt) (NBD-DOPE) were purchased from Avanti Polar Lipids, Inc. (Alabaster, AL, USA). Stearylated octaarginine (STR-R8) was obtained from Kurabo Industries Ltd. (Osaka, Japan). 1,1′-dioctadecyl-3,3,3′,3′-tetramethylindocarbocyanine (DiI) was purchased from MedChemExpress LLC (Monmouth Junction, NJ, USA). rTPA and CAOM were synthesized as previously described [11,18]. HeLa cells were obtained from RIKEN BioResource Research Center (Tsukuba, Japan). Dulbecco’s modified Eagle medium (DMEM) was obtained from FUJIFILM Wako Pure Chemical Corporation (Osaka, Japan). Phosphate-buffered saline (PBS) and the Premix WST-1 Cell Proliferation Assay System were purchased from Takara Bio Inc. (Kusatsu, Japan). Fetal bovine serum (FBS), Singlet Oxygen Sensor Green (SOSG), and MitoTracker Deep Red FM were obtained from Thermo Fisher Scientific (Waltham, MA, USA). Propidium iodide solution was purchased from BioLegend Inc. (San Diego, CA, USA).

### 2.2. Preparation of CAOM-Containing rTPA-MITO-Porters Using iLiNP

**Figure 2:**
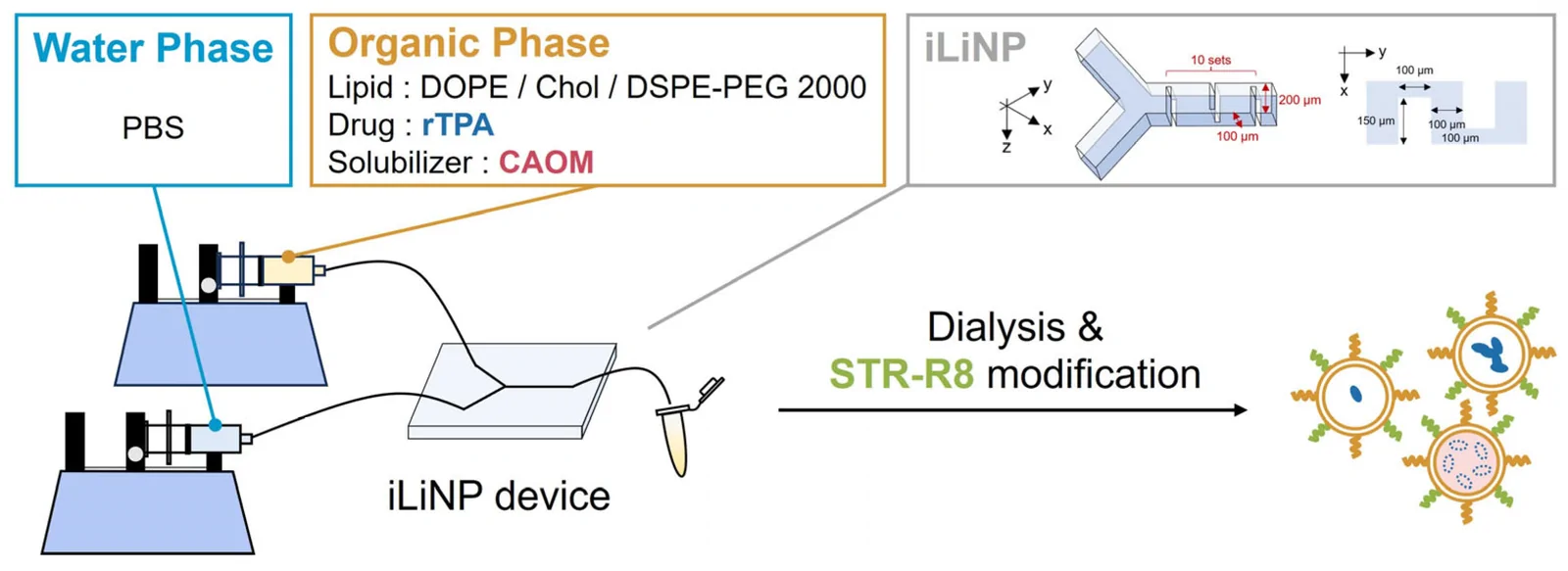
Schematic illustration of rTPA-MITO-Porter preparation using the iLiNP microfluidic device. The aqueous and organic phases were rapidly mixed in the iLiNP device, followed by dialysis and post-modification with STR-R8. CAOM was included in the organic phase for the preparation of High-MP+CAOM.

The invasive Lipid Nanoparticle Production (iLiNP) microfluidic device was used as illustrated in Figure 2 [19]. The organic phase contained 1.3 mM total lipids. Low-MP and High-MP were prepared without CAOM at nominal rTPA-to-total-lipid ratios of 0.5 and 2.5 mol%, respectively. For High-MP+CAOM, the nominal component ratio was DOPE/cholesterol/DSPE-PEG2000/rTPA/CAOM = 9/2/0.22/0.275/88 (molar ratio). The organic-phase components were dissolved in ethanol and rapidly mixed with PBS in the iLiNP device, followed by dialysis against PBS and post-modification with STR-R8. When required for fluorescence analysis, NBD-DOPE or DiI was added at 1.0 mol% of total lipid. Particle size, polydispersity index (PDI), and ζ-potential were measured by dynamic light scattering and electrophoretic light scattering. The total rTPA concentration in the recovered formulation dispersion after dialysis was determined by measuring absorbance at 704 nm after drying and redissolution in DMSO, using an rTPA calibration curve. The assay concentrations were established by dilution based on these measured concentrations. rTPA recovery was calculated relative to the initial amount used for formulation. The nominal rTPA-to-total-lipid ratios refer to the molar ratios of components added during formulation. The total lipid concentrations used in the phototoxicity assays were calculated from formulation inputs and are expressed as nominal concentrations. Detailed preparation and analytical procedures are provided in the Supporting Information.

### 2.3. Evaluation of Singlet Oxygen Generation

Singlet oxygen generation was evaluated using SOSG according to the manufacturer’s instructions. Nanocarrier suspensions were diluted with PBS and mixed with SOSG solution to obtain a total volume of 1000 µL containing 1.0 µM rTPA and 5.0 µM SOSG. The samples were irradiated using a xenon lamp (MAX-303; Asahi Spectra, Tokyo, Japan) at 700 ± 6 nm and 12.5 mW/cm^2^ for 60 s (0.75 J/cm^2^). SOSG fluorescence was measured at 550 nm with excitation at 470 nm using a Varioskan LUX plate reader (Thermo Fisher Scientific, Waltham, MA, USA). Singlet oxygen generation was expressed as *I* − *I*_0_, where *I*_0_ and *I* denote the fluorescence intensities before and after irradiation, respectively.

### 2.4. Flow Cytometric Analysis of Cellular Uptake

HeLa cells were seeded in six-well plates (Corning Inc., Corning, NY, USA) 24 h before the experiment and maintained at 37 °C under 5% CO_2_. After washing with PBS, the cells were incubated for 1 h in serum-free DMEM containing NBD-DOPE-labeled particles. The final total lipid concentration was 27.5 µM. After two washes with PBS containing heparin, the cells were trypsinized and suspended in DMEM containing FBS. After centrifugation, the cell pellet was resuspended in PBS containing bovine serum albumin and sodium azide and analyzed by flow cytometry (CytoFLEX; Beckman Coulter Inc., Brea, CA, USA). Cellular uptake was expressed as the geometric mean fluorescence intensity of NBD-DOPE. Detailed procedures are described in the Supporting Information.

### 2.5. Intracellular Observation Using CLSM

HeLa cells were seeded in 35 mm glass-base dishes (IWAKI Corporation, LTD., Tokyo, Japan) 24 h before the experiment and maintained at 37 °C under 5% CO_2_. After washing, the cells were incubated for 1 h in serum-free DMEM containing DiI-labeled rTPA-MITO-Porter particles at a final total lipid concentration of 27.5 µM. The medium was then replaced with DMEM containing FBS, and the cells were further incubated for 100 min. Mitochondria were stained with MitoTracker Deep Red, after which the cells were washed with PBS, fixed with 4% paraformaldehyde for 30 min, and observed by confocal laser scanning microscopy (CLSM) using a Nikon A1 microscope (NIKON Corporation, Tokyo, Japan). Pearson’s correlation coefficient was calculated within individual whole-cell regions of interest to quantify the spatial intensity correlation between the DiI and MitoTracker fluorescence signals. Detailed procedures are described in the Supporting Information.

### 2.6. Evaluation of Phototoxicity

HeLa cells were seeded in 96-well plates (Greiner Bio-One GmbH, Kremsmünster, Austria) 24 h before treatment. Cells were exposed to the indicated formulations for 1 h in serum-free DMEM and then incubated for 2 h in DMEM containing FBS. After washing, cells were irradiated with a xenon lamp at 700 ± 6 nm and 68.3 mW/cm^2^ for 180 s (12.3 J/cm^2^). WST-1 reagent was added immediately after irradiation, and absorbance was measured after 2 h at 450 nm with 630 nm as the reference wavelength, using a Varioskan LUX plate reader. Cell viability was calculated from *A*_450_ − *A*_630_ and normalized to the nonirradiated PBS-treated control. Detailed procedures are provided in the Supporting Information. The cell-free SOSG assay and the cellular phototoxicity assay used separate irradiation protocols for their different experimental environments and endpoints. Irradiation conditions were identical across formulations within each assay; the two assays were not intended to establish a quantitative correspondence between SOSG fluorescence and cell viability.

### 2.7. Measurement of Fluorescence Spectra

Fluorescence spectra were recorded using a Varioskan LUX plate reader. Nanocarrier suspensions containing 1.0 µM rTPA in PBS were excited at 484 nm, and emission spectra were collected over the wavelength range of 600–800 nm. The maximum fluorescence intensity within this range was used for quantitative comparison.

### 2.8. Cryo-Transmission Electron Microscopy Analysis

Cryo-transmission electron microscopy (cryo-TEM) analysis of the MITO-Porters was performed at Terabase Inc. (Okazaki, Japan). The samples were observed using a JEM-2200FS transmission electron microscope (JEOL Ltd., Tokyo, Japan). Images were acquired using a DE-20 detector (Direct Electron, LP, San Diego, CA, USA) at a specimen temperature of −176 ± 0.5 °C with a defocus value of −3 µm.

Further methodological details for Section 2.2, Section 2.4, Section 2.5 and Section 2.6, together with statistical analysis procedures, are provided in the Supporting Information.

## 3. Results

### 3.1. Preparation and Physicochemical Characterization of CAOM-Containing rTPA-MITO-Porters

Low-MP, High-MP, and High-MP+CAOM were prepared using the iLiNP microfluidic device (Figure 2) [19]. Low-MP and High-MP were prepared using rTPA-to-total-lipid input ratios of 0.5 and 2.5 mol%, respectively, whereas High-MP+CAOM was prepared using an input ratio of 2.5 mol% together with CAOM. As summarized in Table 1, Low-MP and High-MP had mean particle diameters of approximately 70 nm, whereas High-MP+CAOM showed a larger mean diameter of approximately 120 nm. All three formulations exhibited positive ζ-potentials and rTPA recoveries above 70%. High-MP+CAOM showed a lower PDI than Low-MP and High-MP, indicating a narrower particle-size distribution (Figure S1). The coefficient of variation in particle size was also lower for High-MP+CAOM than for the CAOM-free formulations (Table S1).

**Figure 3:**
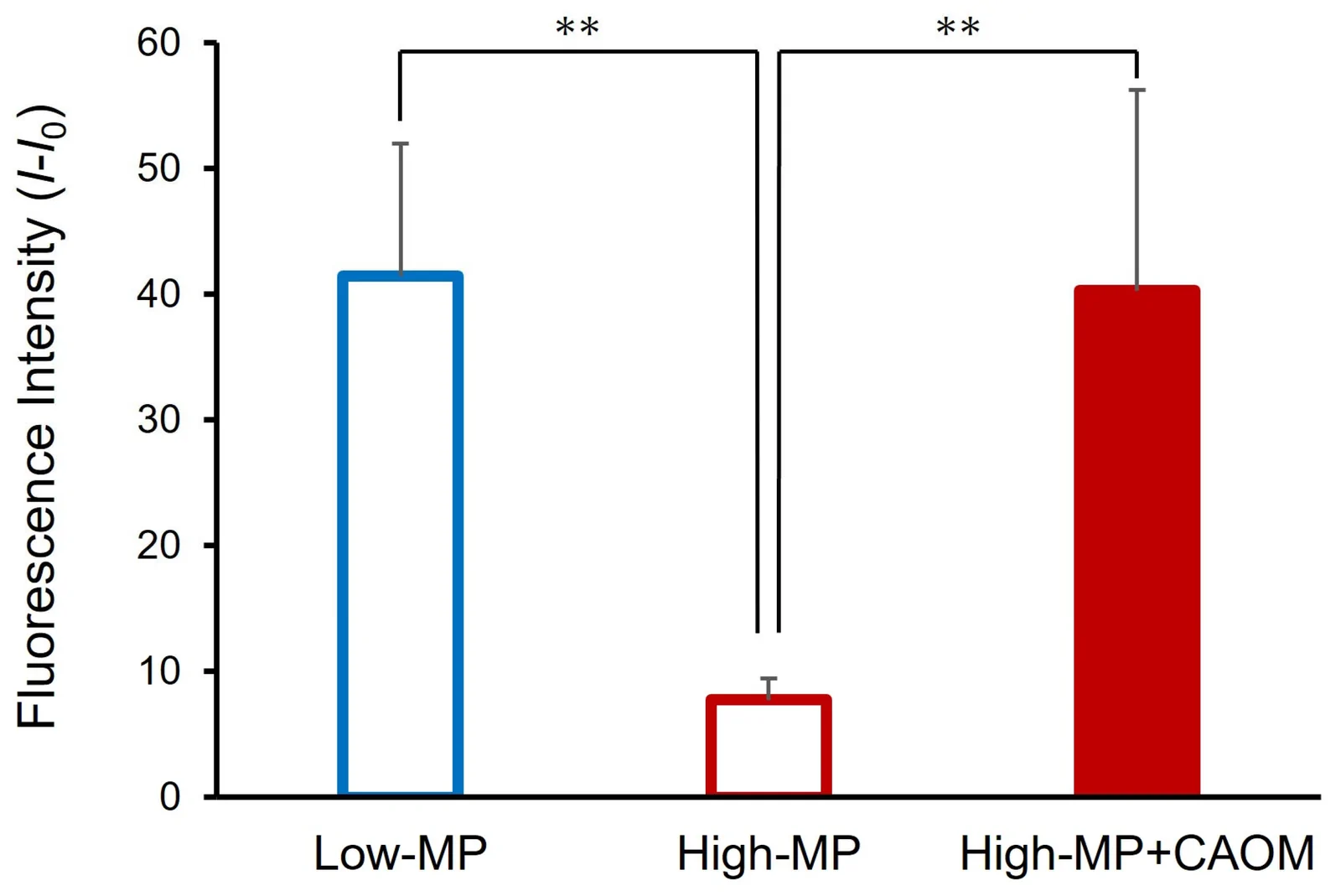
Singlet oxygen generation by rTPA-loaded MITO-Porters. Singlet oxygen generation was evaluated using SOSG at an equivalent rTPA concentration (1.0 µM) in PBS. The change in SOSG fluorescence intensity was expressed as I − I_0_, where I_0_ and I represent the fluorescence intensities before and after light irradiation, respectively. Samples were irradiated using 700 ± 6 nm light at 12.5 mW/cm^2^ for 60 s (0.75 J/cm^2^). Data are presented as mean ± SD (Low-MP, *n* = 5; High-MP, *n* = 8; High-MP+CAOM, *n* = 8). ** *p* < 0.01 by non-repeated-measures ANOVA followed by the Student–Newman–Keuls (SNK) test.

To evaluate the photodynamic properties of the encapsulated rTPA in a cell-free system, singlet oxygen generation was measured using Singlet Oxygen Sensor Green (SOSG). The three formulations were compared at an equivalent rTPA concentration. Singlet oxygen generation was quantified as the difference in SOSG fluorescence intensity before and after light irradiation (I − I_0_). As shown in Figure 3, High-MP exhibited significantly lower singlet oxygen generation than Low-MP. In contrast, High-MP+CAOM showed significantly higher singlet oxygen generation than High-MP, reaching a level comparable to that of Low-MP. These observations support maintenance of the relative SOSG response at high nominal rTPA loading in the CAOM-containing formulation under the tested conditions. They do not identify the molecular origin of this response or establish equivalence between formulations.

### 3.2. Intracellular Behavior of CAOM-Containing rTPA-MITO-Porter

**Figure 4:**
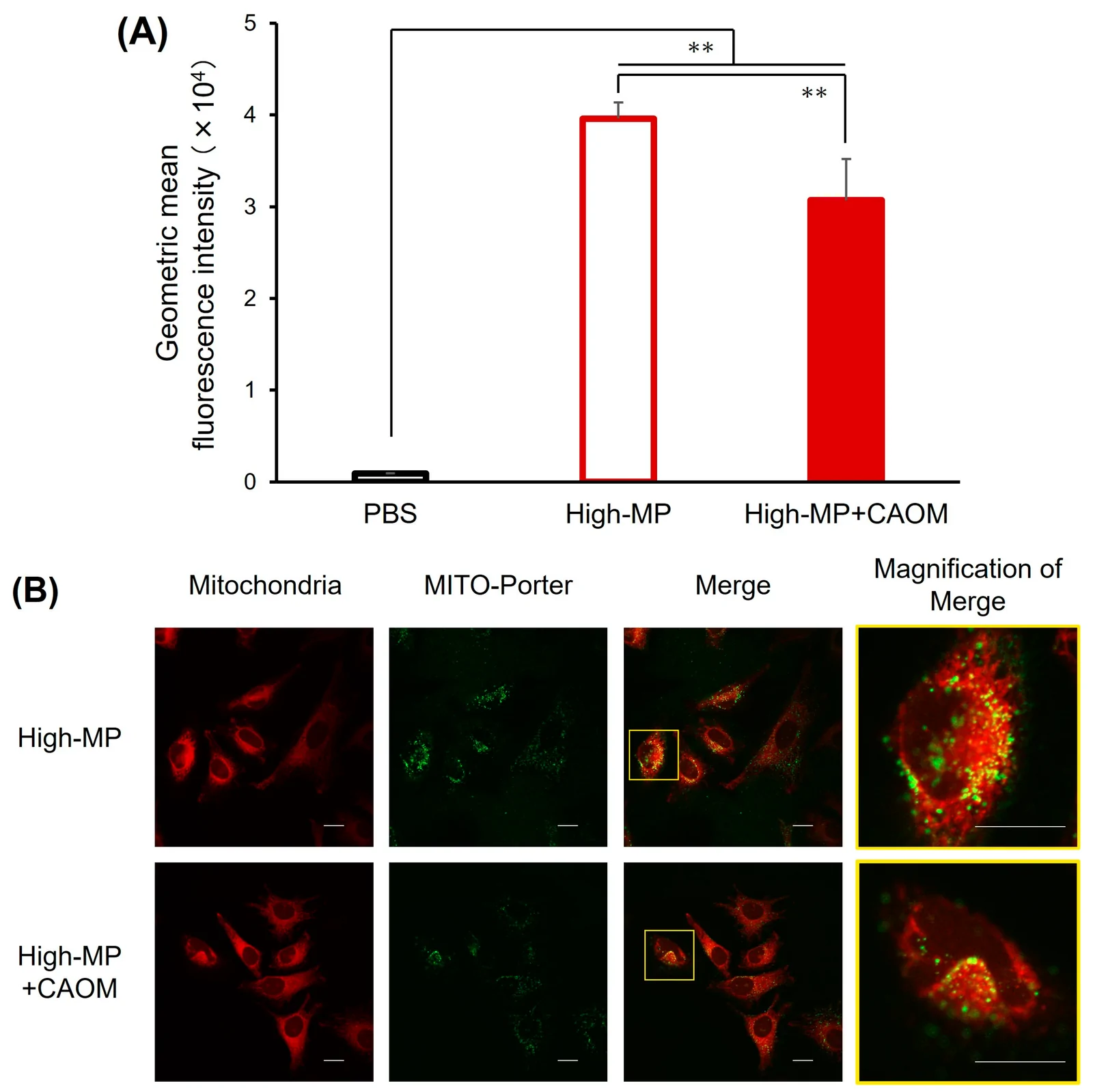
Intracellular trafficking analysis of MITO-Porters in HeLa cells. (**A**) Cellular uptake of High-MP and High-MP+CAOM was evaluated by flow cytometry using NBD-DOPE-labeled particles; PBS-treated cells were used as the control. Cellular uptake was expressed as the geometric mean fluorescence intensity. Data are presented as mean ± SD (PBS, *n* = 4; High-MP, *n* = 3; High-MP+CAOM, *n* = 4). ** *p* < 0.01 by non-repeated-measures ANOVA followed by the SNK test. (**B**) CLSM images of fixed HeLa cells treated with High-MP or High-MP+CAOM. DiI-labeled MITO-Porters are shown in pseudo-green, and mitochondria stained with MitoTracker Deep Red are shown in red. The “Magnification of Merge” images show enlarged views of the regions indicated by the yellow boxes in the “Merged” image. The merged images provide qualitative evidence of spatial proximity and partial overlap between the MITO-Porter and mitochondrial fluorescence signals. Scale bars: 20 µm.

We next examined whether CAOM incorporation affected the cellular uptake and intracellular localization of rTPA-MITO-Porters. Cellular uptake was evaluated by flow cytometry after treatment of HeLa cells with NBD-DOPE-labeled particles for 1 h. As shown in Figure 4A, both High-MP and High-MP+CAOM were internalized by HeLa cells. However, High-MP showed significantly higher cell-associated NBD-DOPE fluorescence than High-MP+CAOM at the same total lipid concentration (*p* < 0.01).

Intracellular localization was subsequently examined by CLSM using DiI-labeled High-MP and High-MP+CAOM. Mitochondria were stained with MitoTracker Deep Red. As shown in Figure 4B, fluorescence signals from both formulations were observed in close proximity to and partially overlapping with mitochondrial signals. Cell-wise Pearson correlation analysis yielded a mean r of 0.464 ± 0.064 for High-MP and 0.419 ± 0.131 for High-MP+CAOM (mean ± SD; Figure S4). These results support positive spatial intensity correlation between the DiI-labeled carrier signal and mitochondrial staining in both formulations.

### 3.3. Photodynamic Cytotoxicity of CAOM-Containing rTPA-MITO-Porter

Before evaluating rTPA-mediated phototoxicity, the effects of the carrier formulations were examined using Empty-MP and Empty-MP+CAOM. Under light irradiation, a reduction in cell viability was observed for Empty-MP+CAOM at the highest tested lipid concentration of 64.0 µM (Figure S2). These results were taken into consideration when selecting the lipid concentrations used for subsequent phototoxicity experiments.

We next compared the three rTPA-containing formulations at an equivalent rTPA concentration to evaluate the photodynamic activity of the encapsulated photosensitizer (Figure S3). Under this condition, High-MP+CAOM did not show an enhanced light-dependent reduction in cell viability compared with the other formulations. Because adjustment to an equivalent rTPA concentration required substantially lower lipid concentrations for High-MP and High-MP+CAOM than for Low-MP, the lower lipid concentration may have resulted in reduced cellular exposure. However, this possibility was not directly evaluated in the present experiment.

**Figure 5:**
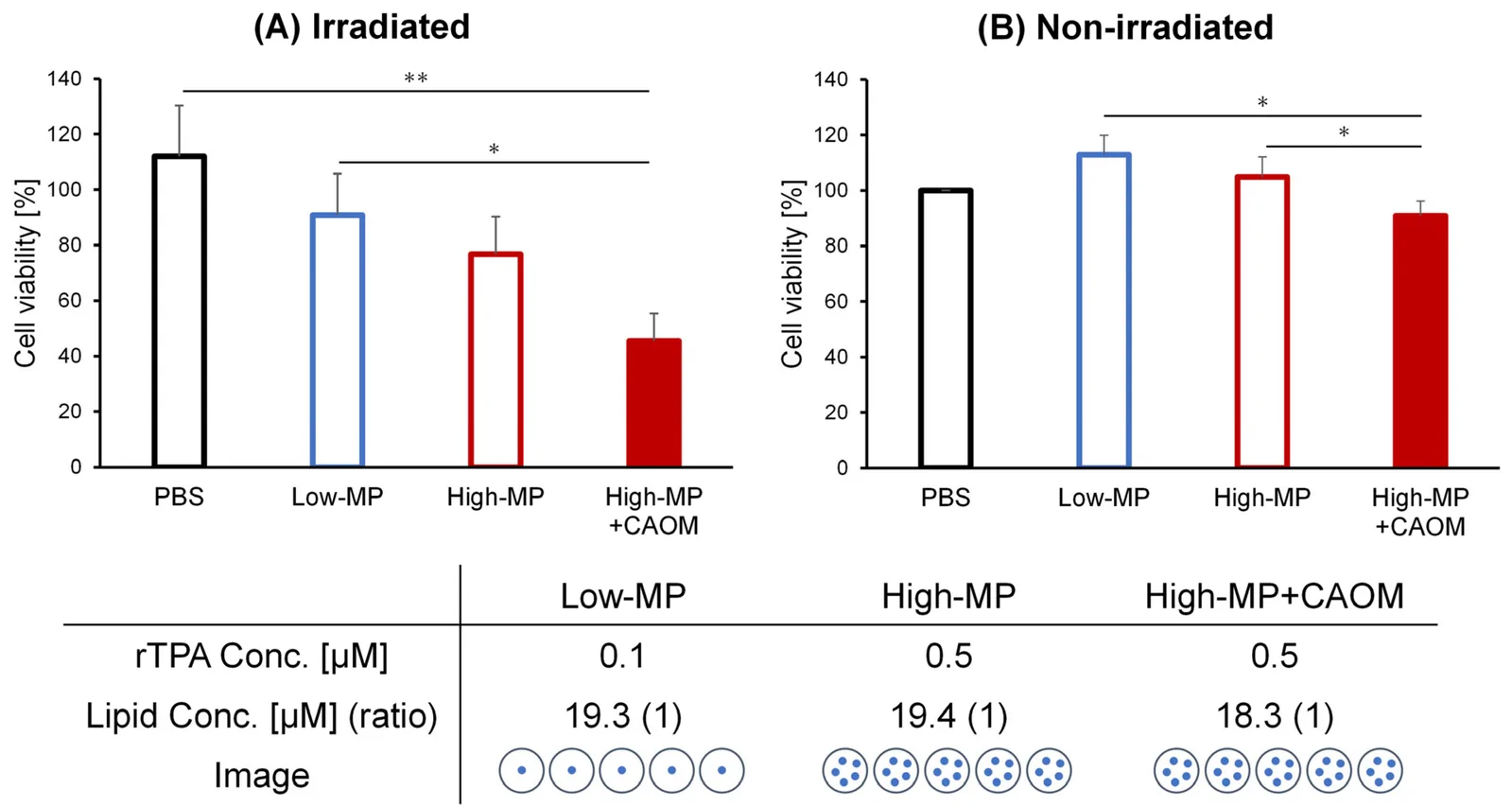
Evaluation of phototoxicity of rTPA-loaded MITO-Porters at comparable total lipid concentrations. HeLa cells were treated with Low-MP, High-MP, or High-MP+CAOM at comparable total lipid concentrations. The rTPA concentrations were 0.1 µM for Low-MP and 0.5 µM for High-MP and High-MP+CAOM, and the corresponding total lipid concentrations were 19.3, 19.4, and 18.3 µM, respectively. The total lipid concentrations were calculated from the lipid amounts used for formulation. Cell viability was determined by the WST-1 assay under (**A**) light-irradiated and (**B**) nonirradiated conditions and normalized to the nonirradiated PBS-treated control, defined as 100%. For panel (**A**), light irradiation was performed using 700 ± 6 nm light at 68.3 mW/cm^2^ for 180 s (12.3 J/cm^2^). For each panel, data are presented as mean ± SD (PBS, *n* = 3; Low-MP, *n* = 3; High-MP, *n* = 3; High-MP+CAOM, *n* = 3). ** *p* < 0.01 and * *p* < 0.05 by non-repeated-measures ANOVA followed by the SNK test.

We therefore compared the formulations at comparable total lipid concentrations to evaluate their photodynamic performance at a comparable lipid dose (Figure 5). Under light irradiation, High-MP+CAOM showed the greatest reduction in mean cell viability among the three formulations. In the absence of light irradiation, cell viability remained close to the control level under the tested conditions. These results indicate that the CAOM-containing formulation exhibited enhanced light-dependent cytotoxicity when the formulations were compared at comparable lipid concentrations.

### 3.4. Fluorescence and Morphological Assessment of Intraparticle rTPA Organization

**Figure 6:**
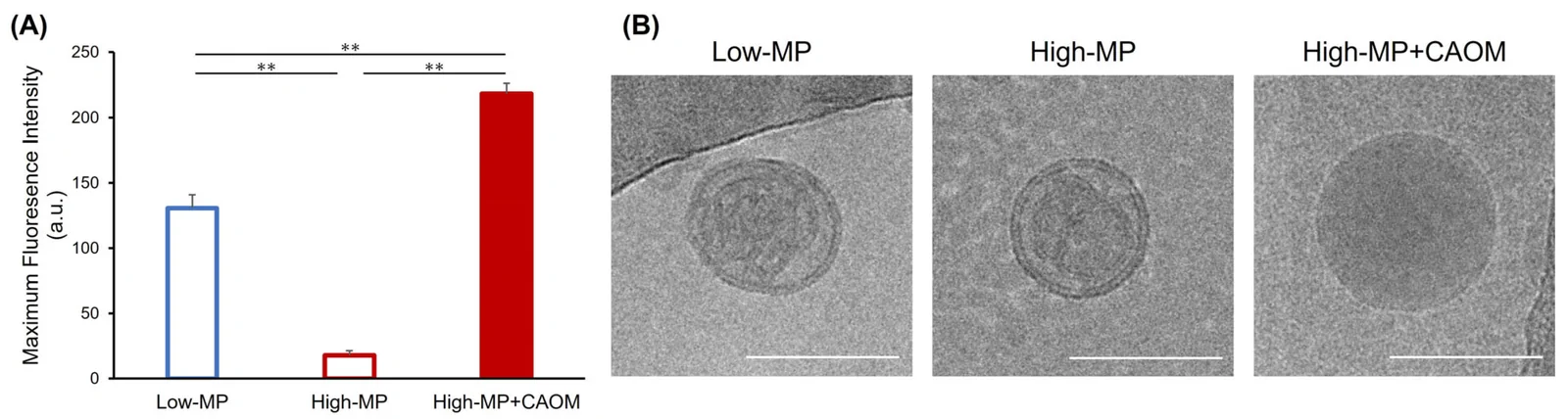
Fluorescence and morphological assessment of rTPA-loaded MITO-Porters. (**A**) Maximum fluorescence intensities of Low-MP, High-MP, and High-MP+CAOM measured in PBS at an equivalent rTPA concentration of 1.0 µM with excitation at 484 nm. The corresponding fluorescence spectra are presented in Figure S5. Data are presented as mean ± SD (Low-MP, *n* = 3; High-MP, *n* = 3; High-MP+CAOM, *n* = 3). ** *p* < 0.01 by non-repeated-measures ANOVA followed by the SNK test. (**B**) Representative cryo-TEM images of Low-MP, High-MP, and High-MP+CAOM showing differences in particle morphology and internal contrast. Scale bars: 200 nm.

To examine whether CAOM incorporation altered the intraparticle organization of rTPA, fluorescence spectra and particle morphology were evaluated. Fluorescence measurements were performed at an equivalent rTPA concentration among the three formulations. As shown in Figure S5, all formulations exhibited an rTPA fluorescence peak near 700 nm. Comparison of the maximum fluorescence intensities showed that High-MP exhibited markedly lower fluorescence than Low-MP, whereas High-MP+CAOM showed significantly higher fluorescence than High-MP (Figure 6A). This pattern is consistent with attenuated ACQ in the CAOM-containing formulation. However, fluorescence intensity alone does not determine the aggregation state of rTPA. Differences in excitation-light absorption, the local environment, or nanoparticle optical properties may also contribute.

Representative cryo-transmission electron microscopy (cryo-TEM) images revealed differences in particle morphology among the formulations (Figure 6B). The CAOM-free formulations showed more heterogeneous internal contrast, whereas High-MP+CAOM exhibited a comparatively homogeneous internal appearance. These observations describe formulation-associated morphological differences. They do not resolve individual rTPA molecules or establish a monomeric state.

## 4. Discussion

rTPA-loaded MITO-Porters were previously developed for mitochondria-targeted PDT. However, increasing the rTPA loading from 0.5 mol% in Low-MP to 2.5 mol% in High-MP did not improve photodynamic performance; instead, High-MP showed lower activity, which was associated with altered intraparticle organization and aggregation of rTPA [17]. In the present study, incorporation of the solubilizer CAOM was associated with fluorescence and SOSG fluorescence responses consistent with attenuated ACQ. At comparable total lipid concentrations, High-MP+CAOM showed a greater light-dependent reduction in cell viability than Low-MP.

The improved photodynamic performance of High-MP+CAOM may be associated with changes in the intraparticle organization of rTPA. CAOM contains aromatic moieties that may interact with hydrophobic aromatic compounds [18], and its incorporation may therefore alter the intermolecular environment of rTPA within the nanoparticles. Direct CAOM–rTPA interactions and the molecular aggregation state were not determined here, so this explanation remains a hypothesis. This interpretation is consistent with previous reports showing that planar photosensitizers are susceptible to ACQ at high local concentrations [12,20].

Consistent with this interpretation, High-MP exhibited markedly lower fluorescence intensity than Low-MP at an equivalent rTPA concentration, whereas incorporation of CAOM significantly increased the fluorescence intensity relative to High-MP (Figure 6A). In addition, High-MP+CAOM showed singlet oxygen generation comparable to that of Low-MP when the formulations were compared at an equivalent rTPA concentration (Figure 3), despite its five-fold higher nominal rTPA-to-lipid ratio. Cryo-TEM further revealed differences in intraparticle morphology between CAOM-free and CAOM-containing formulations (Figure 6B). Taken together, these findings are consistent with attenuated ACQ and the maintenance of photodynamic activity at a high rTPA-to-lipid input ratio. These comparisons describe formulation-level associations but do not establish a quantitative structure–property–function relationship that distinguishes the individual contributions of rTPA loading, CAOM incorporation, and particle properties.

The present study evaluated High-MP+CAOM in both cell-free and cellular systems with the aim of improving formulation-level photodynamic performance at a high rTPA-to-lipid input ratio. At comparable nominal total lipid concentrations, High-MP+CAOM was administered at a higher rTPA concentration than Low-MP and showed a greater light-dependent reduction in cell viability. However, this comparison involved a five-fold difference in rTPA concentration between High-MP+CAOM and Low-MP and does not establish improved cellular photodynamic efficiency per unit rTPA. This formulation-level comparison suggests that administration of a higher rTPA concentration at comparable nominal lipid concentrations can retain photodynamic activity under the tested conditions. However, the present study was restricted to in vitro experiments, and whether these formulation advantages translate into improved antitumor activity in vivo remains to be determined.

High-MP+CAOM showed lower cell-associated NBD-DOPE fluorescence than High-MP in the uptake assay (Figure 4A). Differences in particle size and possible changes in surface organization may influence particle–cell interactions, although their contributions to this difference have not been established. The lipid-label signals in Figure 4 and the cell-free rTPA fluorescence in Figure 6A do not directly determine intracellular rTPA amounts; lower cellular rTPA uptake by High-MP+CAOM therefore cannot be excluded. Nevertheless, High-MP+CAOM produced a greater mean light-dependent reduction in cell viability than High-MP at comparable total lipid concentrations (Figure 5). This phototoxicity result was not accompanied by a higher lipid-label signal in the separate uptake assay. Because the assays used different exposure protocols, these observations do not establish improved photodynamic efficiency per unit of internalized rTPA.

More broadly, ACQ is a common challenge for hydrophobic photosensitizers incorporated at high local concentrations in nanocarriers. The present findings suggest that solubilizer-assisted modulation of intraparticle organization may provide a useful formulation approach for such compounds. Future studies should evaluate whether the effects observed with the rTPA-CAOM combination can be extended to other hydrophobic porphyrin-based or related photosensitizers.

Several limitations of this study should be considered. First, the direct molecular interaction between CAOM and rTPA was not determined; therefore, the proposed modulation of rTPA-rTPA interactions remains an interpretation based on fluorescence, singlet oxygen generation, and morphological observations. Second, cryo-TEM provided information on particle morphology and internal organization but did not directly resolve the molecular aggregation state of rTPA. In addition, CAOM incorporation was accompanied by changes in particle size and PDI, which may influence cellular uptake, intracellular trafficking, and photodynamic activity. The contributions of these particle properties and CAOM incorporation cannot be separated in the present comparison. The DiI images and cell-wise Pearson correlation analysis support spatial association between the DiI-labeled carrier signal and mitochondria but do not establish mitochondrial delivery of rTPA. The contribution of mitochondrial damage to the observed phototoxicity remains to be evaluated. Third, the present study was limited to in vitro evaluation using HeLa cells, and the in vivo performance and safety of the CAOM-containing formulation remain to be investigated. Finally, whether this formulation strategy can be extended to other hydrophobic photosensitizers requires further validation.

## 5. Conclusions

In this study, we developed a CAOM-containing rTPA-MITO-Porter formulation using a nominal rTPA-to-total-lipid input ratio of 2.5 mol% and evaluated its photodynamic performance. CAOM incorporation increased the relative SOSG fluorescence response and fluorescence intensity relative to High-MP at an equivalent rTPA concentration. These observations are consistent with attenuated ACQ but do not directly establish the molecular aggregation state of rTPA or specific CAOM–rTPA interactions. CAOM incorporation was also associated with changes in intraparticle morphology. At comparable nominal total lipid concentrations, High-MP+CAOM exhibited enhanced light-dependent cytotoxicity under the tested conditions. The cellular data demonstrate light-dependent cytotoxicity at a higher administered rTPA concentration than Low-MP under comparable nominal total lipid exposure; they do not demonstrate improved cellular efficiency per rTPA. Further studies are required to evaluate the in vivo performance of this formulation and its applicability to other hydrophobic photosensitizers.

## Figures and Tables

**Table 1:** Physicochemical properties of rTPA-loaded MITO-Porters.

Sample	Particle Size (nm)	PDI	ζ-Potential (mV)	rTPA Recovery (%)
Low-MP	69 ± 11	0.27 ± 0.06	15.4 ± 4.2	76.7 ± 9.3
High-MP	72 ± 9	0.29 ± 0.06	18.1 ± 2.3	77.8 ± 6.3
High-MP+CAOM	122 ± 8	0.09 ± 0.03	20.5 ± 5.2	83.4 ± 4.0

Data are presented as mean ± SD. For particle size and PDI: Low-MP, *n* = 11; High-MP, *n* = 9; High-MP+CAOM, *n* = 10. For ζ-potential: Low-MP, *n* = 6; High-MP, *n* = 5; High-MP+CAOM, *n* = 5. For rTPA recovery: Low-MP, *n* = 4; High-MP, *n* = 4; High-MP+CAOM, *n* = 4. PDI, polydispersity index.

## Data Availability

The original contributions presented in this study are included in the article/Supplementary Materials. Further inquiries can be directed to the corresponding author.

## Supplementary Materials

The following supporting information can be downloaded at: https://www.mdpi.com/article/10.3390/nano16201329/s1, Supplementary Methods; Table S1: Particle size, standard deviation (SD), and coefficient of variation (CV) of rTPA-loaded MITO-Porters; Table S2: Physicochemical properties of Empty-MP and Empty-MP+CAOM; Table S3: Summary of formulation characteristics and cellular observations of rTPA-loaded MITO-Porters; Figure S1: Comparison of polydispersity index (PDI) values of rTPA-loaded MITO-Porters; Figure S2: Evaluation of cytotoxicity of Empty-MP and Empty-MP+CAOM under light irradiation; Figure S3: Evaluation of phototoxicity of rTPA-loaded MITO-Porters at an equivalent rTPA concentration; Figure S4: Pearson correlation analysis of DiI and MitoTracker fluorescence signals in HeLa cells treated with High-MP or High-MP+CAOM; Figure S5: Fluorescence spectra of rTPA-loaded MITO-Porters.

## References

[1] Dougherty T.J., Gomer C.J., Henderson B.W., Jori G., Kessel D., Korbelik M., Moan J., Peng Q. (1998). Photodynamic Therapy. J. Natl. Cancer Inst..

[2] Zhao W., Wang L., Zhang M., Liu Z., Wu C., Pan X., Huang Z., Lu C., Quan G. (2024). Photodynamic therapy for cancer: Mechanisms, photosensitizers, nanocarriers, and clinical studies. MedComm.

[3] Wang X., Peng J., Meng C., Feng F. (2024). Recent advances for enhanced photodynamic therapy: From new mechanisms to innovative strategies. Chem. Sci..

[4] Obaid G., Celli J.P., Broekgaarden M., Bulin A.-L., Uusimaa P., Pogue B., Hasan T., Huang H.-C. (2024). Engineering Photodynamics for Treatment, Priming and Imaging. Nat. Rev. Bioeng..

[5] Zhao Y.-Y., Lu L., Jeong H., Kim H., Li X., Zhang H., Yoon J. (2025). Enhancing biosafety in photodynamic therapy: Progress and perspectives. Chem. Soc. Rev..

[6] Shi C., Huang H., Zhou X., Zhang Z., Ma H., Yao Q., Shao K., Sun W., Du J., Fan J. (2021). Reversing Multidrug Resistance by Inducing Mitochondrial Dysfunction for Enhanced Chemo-Photodynamic Therapy in Tumor. ACS Appl. Mater. Interfaces.

[7] Mahalingam S.M., Ordaz J.D., Low P.S. (2018). Targeting of a Photosensitizer to the Mitochondrion Enhances the Potency of Photodynamic Therapy. ACS Omega.

[8] Ding Q., Rha H., Yoon C., Kim Y., Hong S.J., Kim H.J., Li Y., Lee M.H., Kim J.S. (2025). Regulated cell death mechanisms in mitochondria-targeted phototherapy. J. Control. Release.

[9] Attar G.S., Kumar M., Bhalla V. (2024). Targeting sub-cellular organelles for boosting precision photodynamic therapy. Chem. Commun..

[10] Yamada Y., Akita H., Kamiya H., Kogure K., Yamamoto T., Shinohara Y., Yamashita K., Kobayashi H., Kikuchi H., Harashima H. (2008). MITO-Porter: A liposome-based carrier system for delivery of macromolecules into mitochondria via membrane fusion. Biochim. Biophys. Acta Biomembr..

[11] Satrialdi, Munechika R., Biju V., Takano Y., Harashima H., Yamada Y. (2020). The optimization of cancer photodynamic therapy by utilization of a π-extended porphyrin-type photosensitizer in combination with MITO-Porter. Chem. Commun..

[12] Zhuang Z., Li J., Shen P., Zhao Z., Tang B.Z. (2024). Exploring and Leveraging Aggregation Effects on Reactive Oxygen Species Generation in Photodynamic Therapy. Aggregate.

[13] Sun H., Li L., Guo R., Wang Z., Guo Y., Li Z., Song F. (2024). Suppressing ACQ of Molecular Photosensitizers by Distorting the Conjugated-Plane for Enhanced Tumor Photodynamic Therapy. Chem. Sci..

[14] Ömeroğlu İ., Durmuş M. (2023). Water-soluble phthalocyanine photosensitizers for photodynamic therapy. Turk. J. Chem..

[15] Hamblin M.R. (2020). Photodynamic Therapy for Cancer: What’s Past is Prologue. Photochem. Photobiol..

[16] Satrialdi, Takano Y., Hirata E., Ushijima N., Harashima H., Yamada Y. (2021). An effective in vivo mitochondria-targeting nanocarrier combined with a π-extended porphyrin-type photosensitizer. Nanoscale Adv..

[17] Kubota F., Satrialdi, Takano Y., Maeki M., Tokeshi M., Harashima H., Yamada Y. (2023). Fine-tuning the encapsulation of a photosensitizer in nanoparticles reveals the relationship between internal structure and phototherapeutic effects. J. Biophotonics.

[18] Abbasi S., Sato Y., Kajimoto K., Harashima H. (2020). New Design Strategies for Controlling the Rate of Hydrophobic Drug Release from Nanoemulsions in Blood Circulation. Mol. Pharm..

[19] Kimura N., Maeki M., Sato Y., Note Y., Ishida A., Tani H., Harashima H., Tokeshi M. (2018). Development of the iLiNP Device: Fine Tuning the Lipid Nanoparticle Size within 10 nm for Drug Delivery. ACS Omega.

[20] Gorbunova V., Shi L., Nguyen C., Gary-Bobo M., Richy N., Paul F., Mongin O., Cammas-Marion S., Paul-Roth C.O. (2026). Encapsulation of a Hydrophobic Fluorenyl-Porphyrin in Biocompatible Nanoparticles: Influence of Photosensitizer Loadings on Two-Photon Phototherapeutic Potential. ChemistryEurope.

