# Supplementary material for: Solubilizer-Assisted Formulation of Porphyrin-Containing Lipid Nanoparticles for Photodynamic Applications
Source: Nanomaterials (Basel). 2026 Oct 20;16(20):1329. doi: 10.3390/nano16201329 (PMC13708445; doi:10.3390/nano16201329)
Supplement: Supplementary file 1 [file nanomaterials-16-01329-s001.zip › nanomaterials-4547582-supplementary.pdf]

SUPPLEMENTARY INFORMATION

Supplementary Methods

Preparation of CAOM-Containing rTPA-MITO-Porters Using iLiNP

The invasive Lipid Nanoparticle Production (iLiNP) microfluidic device contained 10 baffle mixer sets. The standard dimensions of each baffle mixer were a width of 150 μm, a depth of 100 μm, and an interval of 100 μm. The organic phase contained 1.3 mM total lipids. Low-MP and High-MP were prepared without CAOM at nominal rTPA-to-total-lipid ratios of 0.5 mol% (X = 0.055) and 2.5 mol% (X = 0.275), respectively. For High-MP+CAOM, the nominal component ratio was DOPE/cholesterol/DSPE-PEG2000/rTPA/CAOM = 9/2/0.22/0.275/88 (molar ratio). When fluorescent labeling was required, NBD-DOPE or DiI was added at 1.0 mol% of total lipid.

The organic phase was rapidly mixed with PBS using the iLiNP device at a combined flow rate of 500 μL/min, with flow rates of 150 μL/min for the organic phase and 350 μL/min for PBS, corresponding to a final ethanol concentration of 30%. Syringe pumps (Harvard Apparatus, Holliston, MA, USA) were used to control the flow rates. The resulting suspension was dialyzed against PBS for at least 2 h using Spectra/Por 4 dialysis membranes (molecular weight cutoff, 12,000–14,000 Da; Spectrum Laboratories, Inc., Rancho Dominguez, CA, USA). After dialysis, STR-R8 was post-modified at 10 mol% relative to total lipid.

Particle size, polydispersity index (PDI), and ζ-potential were measured using a Zetasizer Nano ZS (Malvern Panalytical, Worcestershire, UK). Particle size and PDI were

determined by dynamic light scattering, and ζ-potential was determined by electrophoretic light scattering.

The total rTPA concentration in the recovered formulation dispersion after dialysis was determined by measuring absorbance at 704 nm after drying and redissolution in DMSO, using an rTPA calibration curve. The assay concentrations of 0.1, 0.5, and 1.0 μM were established by dilution based on these measured concentrations.

rTPA recovery (%) = [amount of rTPA recovered in the nanoparticle suspension (mol) / initial amount of rTPA used for formulation (mol)] × 100

Flow Cytometric Analysis of Cellular Uptake

HeLa cells (2 mL, 1 × 10^5 cells/mL) were seeded in 6-well plates (Corning Inc., NY, USA) 24 h before the experiment and incubated at 37°C under 5% CO_2. After washing with 1 mL of PBS, the cells were incubated for 1 h in serum-free DMEM containing NBD-DOPE-labeled rTPA-MITO-Porter particles at a final total lipid concentration of 27.5 μM. After two washes with 500 μL of PBS containing heparin (20 U/mL), the cells were trypsinized and suspended in DMEM containing FBS. Following centrifugation at 700 × g for 3 min at 4°C, the supernatant was removed, and the cell pellet was resuspended in PBS containing 0.5% bovine serum albumin and 0.1% sodium azide.

The cells were stained with propidium iodide (PI; final concentration, 5 μg/mL) for 15 min at 4°C in the dark. The cell suspension was passed through a nylon mesh and analyzed using a CytoFLEX flow cytometer (Beckman Coulter Inc., Brea, CA, USA). NBD-DOPE and PI were excited at 488 nm. NBD-DOPE fluorescence was collected using a 525/40 nm band-pass filter, and PI fluorescence was collected using a 585/42 nm

band-pass filter. Spectral spillover between the two fluorophores was compensated using single-stained controls. Cellular uptake was evaluated in PI-negative cells and expressed as the geometric mean fluorescence intensity of NBD-DOPE.

Intracellular Observation by Confocal Laser Scanning Microscopy (CLSM)

HeLa cells (2 mL, 5 × 10^4 cells/mL) were seeded in 35 mm glass-base dishes (IWAKI Corporation, LTD., Tokyo, Japan) 24 h before the experiment and incubated at 37°C under 5% CO_2. After washing with 1 mL of serum-free DMEM, the cells were incubated for 1 h in serum-free DMEM containing DiI-labeled rTPA-MITO-Porter particles at a final total lipid concentration of 27.5 μM. The medium was then replaced with 1 mL of fresh DMEM containing FBS, and the cells were further incubated for 100 min. Mitochondria were stained with MitoTracker Deep Red (final concentration, 100 nM) for 30 min. The cells were then washed with PBS and fixed with 4% paraformaldehyde for 30 min. After fixation, the cells were washed with PBS and observed by CLSM.

CLSM images were acquired using a Nikon A1 microscope (NIKON Corporation, Tokyo, Japan) equipped with a water-immersion objective lens (Plan Apo VC 60× WI DIC N2) and a dichroic mirror (DM405/488/561/640). DiI and MitoTracker Deep Red were excited at 561 and 638 nm, respectively. The fluorescence detection channels were set to 595/50 nm for DiI and 700/75 nm for MitoTracker Deep Red.

Pearson Correlation Analysis

Original single-plane Nikon A1 ND2 images were analyzed using Python and NumPy. Whole-cell regions of interest, including nuclear regions, were manually

delineated. Cells with incomplete or ambiguous boundaries were excluded, and repeated observations of the same cell were counted only once. Pearson's correlation coefficient (r) was calculated separately for each cell from paired raw DiI and MitoTracker pixel intensities within the region of interest, without background subtraction, intensity thresholding, smoothing, or intensity rescaling. The analysis included 15 unique cells for High-MP and 9 unique cells for High-MP+CAOM. Group summaries were calculated as the arithmetic mean and sample standard deviation of the individual cell coefficients. Independent biological replicate identities could not be established from the available records; therefore, the analysis was descriptive and no inferential comparison between formulations was performed.

Some pixels reached the detector maximum of 4095. In a separate sensitivity analysis, exclusion of pixel pairs in which either channel reached this maximum changed each group mean r by less than 0.001. The primary results were calculated from the unmodified raw pixel values.

Evaluation of Phototoxicity

HeLa cells (100 μL, 3×10^4 cells/mL) were seeded in 96-well plates (Greiner Bio-One GmbH, Kremsmünster, Austria) 24 h before the experiment and incubated at 37°C under 5% CO_2. After washing with 100 μL of PBS, the cells were treated with 100 μL of the indicated samples in serum-free DMEM for 1 h. The medium was then replaced with DMEM containing FBS, and the cells were further incubated for 2 h.

After washing with 200 μL of PBS, 100 μL of DMEM containing FBS was added, and the cells were irradiated using a xenon lamp at 700 ± 6 nm and 68.3 mW/cm² for 180 s (12.3 J/cm²). Immediately after irradiation, WST-1 reagent was added to DMEM

containing FBS at a 1:10 ratio (final volume, 100 μL). The cells were incubated for 2 h at 37°C under 5% CO_2. Absorbance was measured at 450 nm with 630 nm as the reference wavelength using a Varioskan LUX plate reader (Thermo Fisher Scientific, Waltham, MA, USA). Cell viability was calculated from A450 − A630 and expressed relative to the nonirradiated PBS-treated control, defined as 100%. Sample concentrations used in each experiment are indicated in the corresponding figure legends.

Statistical Analysis

Data are presented as the mean ± standard deviation (SD). Statistical comparisons among multiple groups were performed using non-repeated-measures analysis of variance (ANOVA), followed by the Student–Newman–Keuls (SNK) post hoc test. A p-value < 0.05 was considered statistically significant. The exact sample size (n) for each experimental group is indicated in the corresponding figure or table legend.

For the Pearson correlation analysis, n denotes the number of unique analyzed cells, and no inferential comparison between formulations was performed.

Supplementary Tables

Table S1. Particle size, standard deviation (SD), and coefficient of variation (CV) of rTPA-loaded MITO-Porters.

Sample	Particle size (nm)	SD (nm)	CV
Low-MP	69	11	0.15
High-MP	72	9	0.13
High-MP+CAOM	122	8	0.07

Particle-size measurements were obtained independently for each formulation (Low-MP, n = 11; High-MP, n = 9; High-MP+CAOM, n = 10). The mean particle sizes and SD values correspond to those presented in Table 1 of the main text.

Table S2. Physicochemical properties of Empty-MP and Empty-MP+CAOM.

Formulation	Particle size (nm)	PDI	ζ-potential (mV)
Empty-MP	72 ± 12	0.28 ± 0.05	11.8 ± 4.7
Empty-MP+CAOM	124 ± 10	0.14 ± 0.06	21.2 ± 2.0

Data are presented as mean ± SD (Empty-MP, n = 4; Empty-MP+CAOM, n = 5). PDI, polydispersity index.

Table S3. Summary of formulation characteristics and cellular observations of rTPA-loaded MITO-Porters.

Formulation	rTPA/Lipid (mol%)	CAOM	Fluorescence quenching tendency	Cellular uptake	Mitochondrial proximity
Low-MP	0.5	−	Lower	Previously observed [17]	Previously observed [17]
High-MP	2.5	−	Higher	Observed	Observed
High-MP+CAOM	2.5	+	Attenuated relative to High-MP	Observed	Observed

The rTPA/lipid values (mol%) represent the nominal molar input ratios used for formulation. Fluorescence-quenching tendency is inferred from fluorescence measurements and is not a direct measure of rTPA aggregation. “Mitochondrial proximity” summarizes fluorescence-microscopy observations. For High-MP and High-MP+CAOM, these observations are supplemented by Pearson correlation analysis (Figure S4), which does not establish mitochondrial delivery of rTPA.

Supplementary Figures

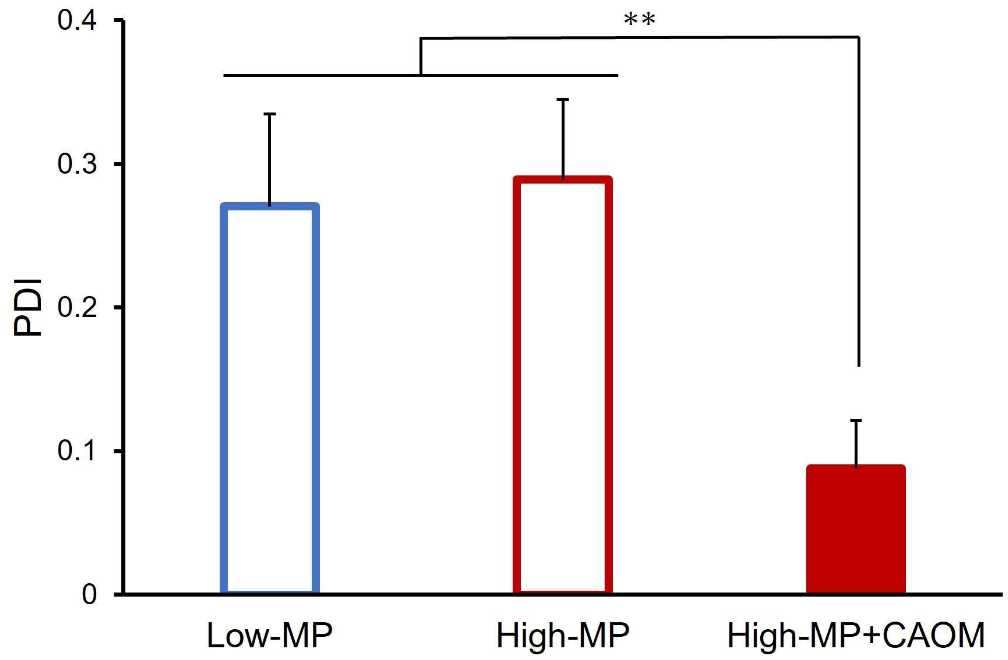

Figure S1. Comparison of polydispersity index (PDI) values of rTPA-loaded MITO-Porters. Data are presented as mean ± SD (Low-MP, n = 11; High-MP, n = 9; High-MP+CAOM, n = 10). **$p < 0.01$ by non-repeated-measures ANOVA followed by the SNK test.

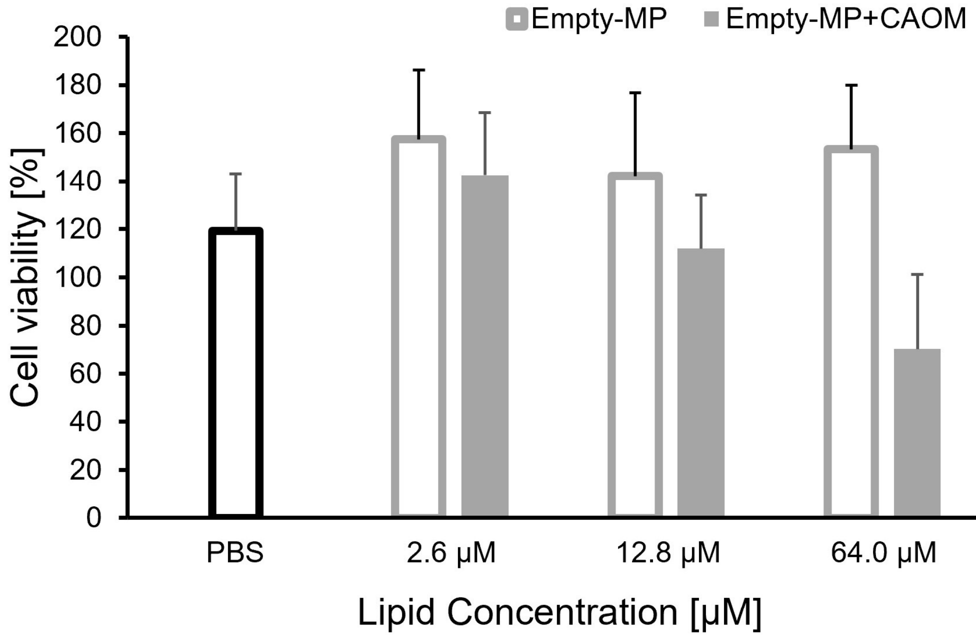

Figure S2. Evaluation of cytotoxicity of Empty-MP and Empty-MP+CAOM under light irradiation. HeLa cells were treated with Empty-MP or Empty-MP+CAOM at total lipid concentrations of 2.6, 12.8, and 64 µM and irradiated using 700 ± 6 nm light at 68.3 mW/cm² for 180 s (12.3 J/cm²). Cell viability was determined by the WST-1 assay and normalized to the PBS-treated control without light irradiation, defined as 100%. Data are presented as mean ± SD (PBS, n = 4; Empty-MP, 2.6 µM: n = 5; Empty-MP, 12.8 µM: n = 6; Empty-MP, 64.0 µM: n = 6; Empty-MP+CAOM, 2.6 µM: n = 4; Empty-MP+CAOM, 12.8 µM: n = 4; Empty-MP+CAOM, 64 µM: n = 4).

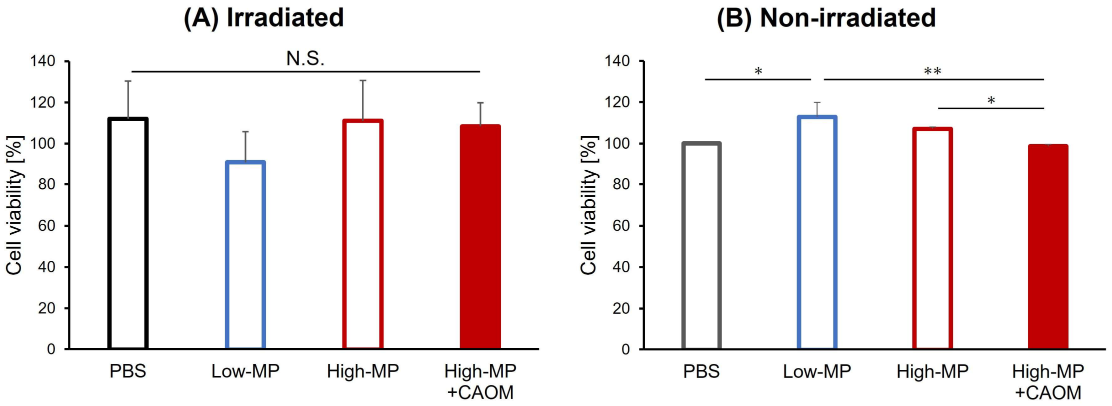

	Low-MP	High-MP	High-MP+CAOM
rTPA Conc. [µM]	0.1	0.1	0.1
Lipid Conc. [µM] (ratio)	19.3 (5)	3.9 (1)	3.7 (1)
Image	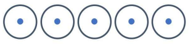	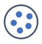	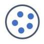

Figure S3. Evaluation of phototoxicity of rTPA-loaded MITO-Porters at an equivalent rTPA concentration. HeLa cells were treated with Low-MP, High-MP, or High-MP+CAOM at an equivalent rTPA concentration of 0.1 µM. The corresponding total lipid concentrations were 19.3 µM for Low-MP, 3.9 µM for High-MP, and 3.7 µM for High-MP+CAOM. The total lipid concentrations were calculated from the lipid amounts used for formulation. Cell viability was determined by the WST-1 assay under (A) light-irradiated and (B) nonirradiated conditions and normalized to the PBS-treated control without light irradiation, defined as 100%. For panel (A), light irradiation was performed using 700 ± 6 nm light at 68.3 mW/cm² for 180 s (12.3 J/cm²). Data are presented as mean ± SD (PBS, n = 3; Low-MP, n = 3; High-MP, n = 3; High-MP+CAOM, n = 3). **$p < 0.01$, *$p < 0.05$; N.S., not significant, by non-repeated-measures ANOVA followed by the SNK test.

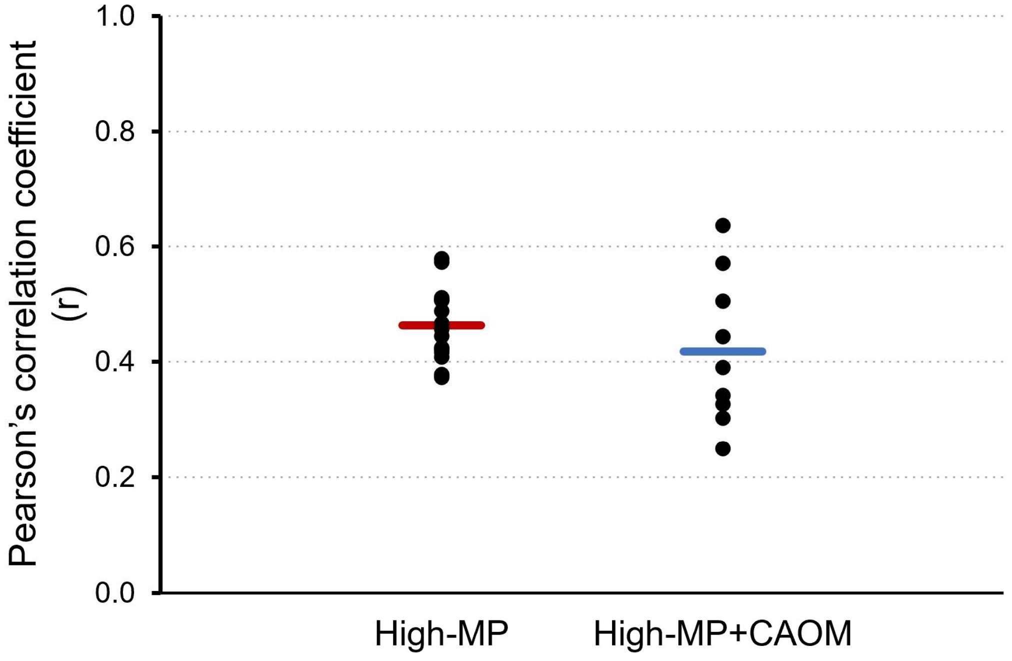

Figure S4. Pearson correlation analysis of DiI and MitoTracker fluorescence signals in HeLa cells treated with High-MP or High-MP+CAOM. Pearson's correlation coefficient was calculated within individual whole-cell regions of interest using the original CLSM images. Each point represents one unique cell; repeated observations of the same cell were excluded. The mean ± SD was 0.464 ± 0.064 for High-MP (n = 15 cells) and 0.419 ± 0.131 for High-MP+CAOM (n = 9 cells). Results are presented descriptively, without inferential comparisons between formulations.

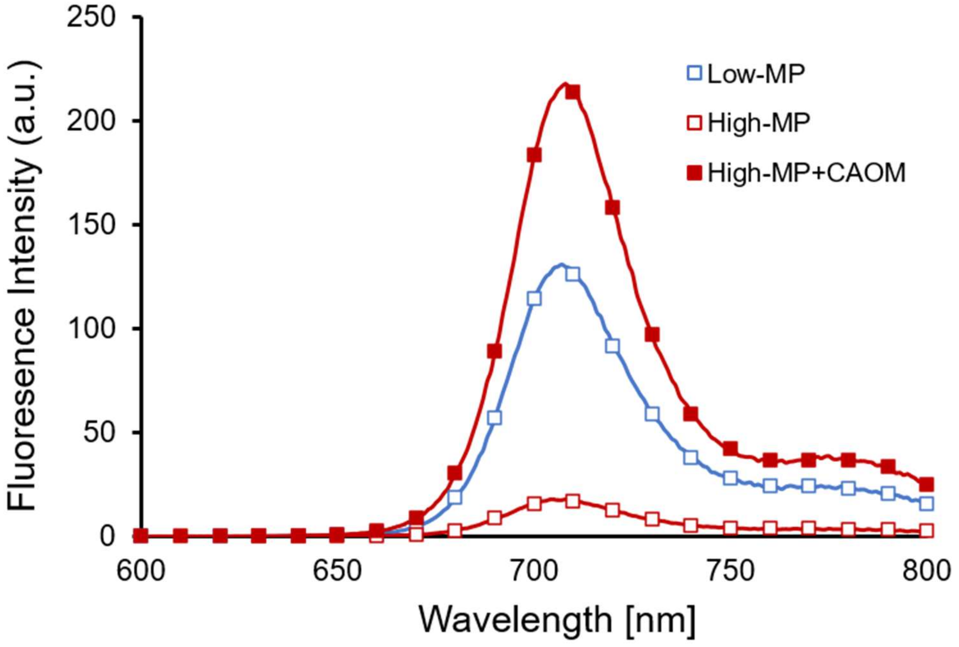

Figure S5. Fluorescence spectra of rTPA-loaded MITO-Porters. Fluorescence spectra of Low-MP, High-MP, and High-MP+CAOM were measured in PBS at an equivalent rTPA concentration of 1.0 μM with excitation at 484 nm. Data are presented as mean (Low-MP, n = 3; High-MP, n = 3; High-MP+CAOM, n = 3).
